# Submovement control processes in discrete aiming as a function of space-time constraints

**DOI:** 10.1371/journal.pone.0189328

**Published:** 2017-12-27

**Authors:** Tsung-Yu Hsieh, Yeou-Teh Liu, Karl M. Newell

**Affiliations:** 1 National Taiwan Normal University, Taipei, Taiwan; 2 University of Georgia, Athens, Georgia, United State of America; Nanyang Technological University, SINGAPORE

## Abstract

There is preliminary evidence that there are several types of submovements in movement aiming that reflect different processes of control and can result from particular task constraints. The purpose of the study was to investigate the effect of movement space and time task criteria on the prevalence of different submovement control characteristics in discrete aiming. Twelve participants completed 3 distance x 5 time conditions each with 100 trials in a target-aiming movement task. The kinematic structure of the trajectory determined the prevalence of 5 submovement types (none; pre-peak, post-peak movement velocity; undershoot, overshoot). The findings showed that the overall number of submovements increased in the slower space-time conditions and was predominantly characterized by post-peak trajectory submovements rather than discrete overshoot submovements. Overshoot submovements were more frequent in the high average movement velocity and short time duration conditions. We concluded that there are qualitatively different distributional patterns of submovement types in discrete aiming tasks that are organized by the quantitative scaling of the average movement velocity arising from multiple control processes to meet the specific space-time task constraints.

## Introduction

There are many manual tasks where a high degree of accuracy and efficiency of control is needed in aiming a movement at a target. Completing movement-aiming tasks more quickly than usual typically results in an increase in spatial error. This is the classic phenomenon of the trade-off between movement speed and accuracy that is a fundamental and long-standing problem in the field of motor control [1–4].

Since Woodworth’s [4] seminal study of the accuracy of voluntary movement the role of submovements during aiming tasks has been investigated to reveal the control of the accuracy of human movement [5–9]. Woodworth [4] proposed that discrete aiming movements consisted of two successive phases that he called initial adjustment and current control, respectively. His landmark investigation distinguished the role of a current control phase based on the change in the kinematic trajectory of movement and showed that it was a significant factor that related to movement accuracy.

**Fig 1:**
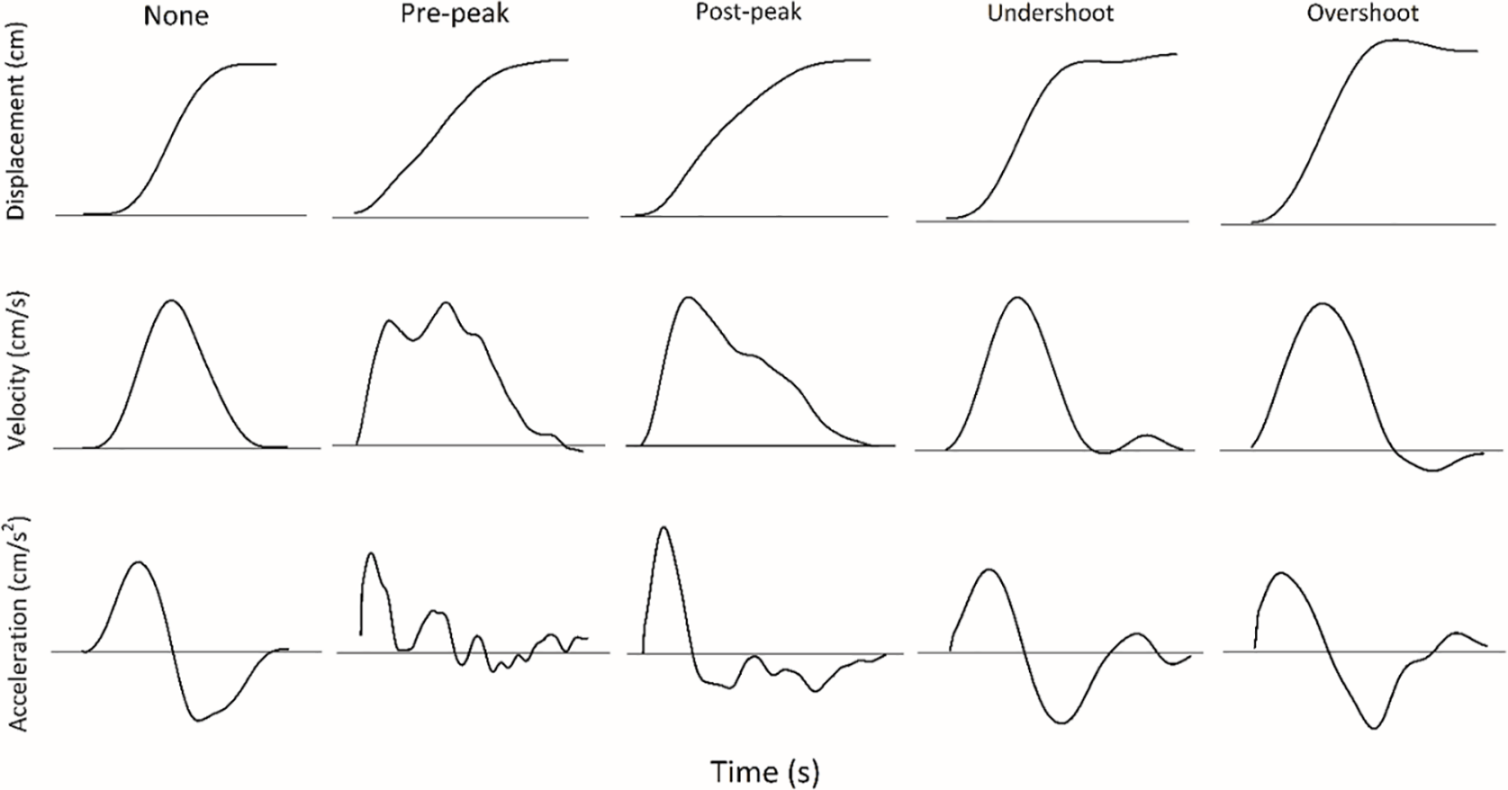
Example of four types of submovements at 20 cm as defined by Chua and Elliott [6]’s algorithm. The different columns indicate types of submovement in displacement, velocity and acceleration profiles (from left to right: none, pre-peak velocity, post-peak velocity, undershoot, and overshoot). The solid line indicates zero level for the respective variable.

Aiming movements often consist of small discrete phases or irregularities that are expressed in terms of submovements. The characterization of submovements in the Woodworth [4] model has motivated attempts to express submovement structure in terms of movement control theories [3, 10–12]. The classic approach was that of Crossman and Goodeve [10] who proposed an iterative correction model based on the feedback theory whereby an aiming movement was composed of a series of ballistic submovements (e.g., undershooting and overshooting). Based on their model, each submovement was assumed to have a similar duration and the movement error associated with remaining distance. Undershooting was the primary submovement that falls short of the target, then the secondary movement would hit the target; and overshooting was the primary submovement that overshoots the target, then the reverse movement (second submovement) would hit the target (see Fig 1). The subsequent submovements were proposed to be due to visual information and other feedback obtained from the variability of a current or previous submovement. Without vision, the corrective process has been taken to be based on proprioceptive information to make the discrete submovement [1, 6, 9].

According to Crossman and Goodeve [10], visual information was the main factor in controlling the submovements in aiming tasks (see also [6, 13–15]). However, the feedback explanation has been challenged as a general account of the movement speed-accuracy trade-off given that the time to obtain and process information is often too long for the control of short duration rapid aiming movements [16]. In light of this interpretation, feedforward processes of limb control have been postulated where the movement trajectory is determined from a motor program before a movement begins [3, 12, 16–17].

In addition to the contrasting feedback and feedforward mechanisms of control characteristics, a visuo-motor control process, such as the optimized-submovement model, has been proposed through the analysis of the kinematics of movement trajectories [9]. This model holds that execution of the initial impulse or primary submovement is affected by neural noise in the motor system [3]. In this case, it has been assumed that corrective submovements only occur once the primary submovement is anticipated to miss the target (either undershooting or overshooting), and this increases the overall movement time.

Traditionally, submovements in the final portion of the discrete movement have been viewed as movement corrections. In addition, Chua and Elliott [6] proposed that initial adjustment (e.g., [4]) might not appear to be as ballistic as interpreted in past studies. They offered the continuous control point of view and suggested that significant deviation from acceleration profile might infer a gradual decease of braking force of active limb toward the target without velocity increasing as opposed to discrete correction. A considerable amount of evidence for the above explanation has been provided by the observation that increases in the number of submovements usually result from narrowing the target size under the instruction to participants to move as fast and accurately as possible [1, 9–10]. However, Fradet, Lee, and Dounskaia [18] have questioned the traditional correction interpretation of the role of submovements by pointing out that a secondary movement may emerge during motion termination and during a low velocity movement by decreases in target size. They suggested that many of the submovements arising from biomechanical sources of movement variability may not be corrective fluctuations but rather can be related to task constraints [19].

By manipulating the type of movement task (discrete, reciprocal, and passing) and target size (small and large), they investigated the contribution of different task factors to submovement production. It was found that there were three types of submovement across the tasks that also held relations to the subcorrection framework of Chua and Elliot [6]. What Dounskaia et al. [19] called type 1 submovements were defined as a zero crossing from positive to negative value that occurred in a single velocity profile; type 2 were defined as a zero crossing from negative to positive value that occurred in the acceleration profile; and type 3 were defined as a zero crossing from positive to negative value that occurred in the jerk profile (e.g., the criteria for identifying a submovement were standard for different investigators, but the identifications of type of submovement is different from research groups; Dounskaia et al. [19] suggested that type 1 is related to overshooting, type 2 is undershooting, and pre and post peak submovement corresponded to type 3). Their results showed that production of submovements during an aiming movement was influenced by different task factors. They found that type 1 submovements tended to emerge due to motion termination during the discrete and reciprocal tasks at the small targets [19–20]; type 2 submovements were related either to motion termination or accuracy regulation; and type 3 submovements were related to motion fluctuations when movement speed decreases (e.g., [8]).

Although the spatial precision requirement in aiming movements is accompanied by the more frequent emergence of corrective submovements, high temporal precision requirements also influence the characteristics of submovement structure [3, 21]. Carlton [22] provided kinematic data to support this temporal hypothesis for discrete movement aiming. He showed that temporal precision tasks are characterized by a single acceleration and deceleration phase even though the movement time was as long as 400 ms and visual feedback was available through the whole movement trajectory. Moreover, visual regulation may proceed in a continuous fashion rather than discrete corrective strategy [6, 23].

According to these findings, the characteristics and prevalence of submovements can result from different task constraints [19, 22, 24]. Most of the previous studies have focused on the condition that only instructed participants to move as fast as possible in a time minimization task (e.g., Fitts’ paradigm) [6, 9, 14, 18, 25–27]. Only a few submovement studies have instructed participants to deliberately control the duration of the criterion movement time [8, 28–29]. However, the accuracy and variability of movement spatial and temporal error in movement speed and accuracy are influenced by the interaction of the movement properties of amplitude, time and the emergent average velocity [24, 30].

The purpose of present study was to examine the effects of movement amplitude and movement time on the prevalence of particular characteristic submovements by systematically manipulating a broad range of movement space and time criteria in a discrete movement-aiming task. Two primary hypotheses were tested: 1) the number of submovements (independent of type) will be fewer or even non-existent in short duration and/or high average movement velocity conditions (e.g., [3]), and 2) the number of submovements that occur pre and post peak velocity (e.g., [11]) will increase from middle average velocity through to slow movement velocity conditions. Experimental support of these hypotheses would provide evidence for the differential qualitative engagement of multiple control processes in discrete movements (e.g., [31]) as a function of the space-time task constraints.

## Methods

For the present study, we reanalyzed the dataset of a previously published experiment [24] that had a wide range of movement amplitude-time conditions. The original paper has a detailed description of the methods so that we report them here briefly. The details of movement outcome (e.g., movement time, spatial error etc.) were reported in Hsieh, Pacheco, and Newell [24].

### Participants

Twelve self-reported right-handed healthy young adults (6 males and 6 females) volunteered for the experiment (aged: 28.17 ± 3.58 yr). All participants read and signed informed consent before participating in the experiment and the Institutional Review Board of Penn State University approved the experimental procedures.

### Apparatus

A Wacom Cintiq 21UX digital tablet (Model DTZ-2100D, 561 x 421 x 61.3 mm with an active surface area of 432 mm x 324 mm) was connected to a PC computer (the pixel range was set at 800 x 600) and a handheld stylus (Model ZP-501E) was used for data collection. A customized program running a discrete aiming task protocol was used to adjust different criteria of movement time and amplitude goals in space-time conditions. The actual distance moved by the stylus on the tablet corresponding to the distance moved by the cursor was 1:1. The position of the stylus was sampled at 130 Hz.

### Experimental design

Participants performed discrete aiming movements over 3 different movement amplitudes (10, 20, and 30 cm). The target movement times ranged from 10 cm (fast: 250 ms, fast-middle: 300 ms, middle: 550 ms, middle accurate: 1000 ms, and accurate: 1300 ms), 20 cm (fast: 300 ms, fast-middle: 450 ms, middle: 650 ms, middle accurate: 1500 ms, and accurate: 2000 ms), and 30 cm (fast: 350 ms, fast-middle: 550 ms, middle: 750 ms, middle accurate: 1800 ms, and accurate: 2500 ms). The details of experimental design are provided elsewhere [24].

Each participant completed 3 distance x 5 time conditions each with 100 trials of a discrete aiming task. It took approximately 1 hr to complete the 5 space-time conditions on each day. Each participant participated in the study for 3 days to complete the 15 testing conditions. We randomly determined the order of the 5 conditions within a day and the order of amplitudes over days for each participant.

### Procedures

The task was to slide a stylus from left (start position: 2 mm in diameter) to stop on the right (target position: 1 mm in diameter) in the target time. The digital tablet was positioned at the middle and in front of the participant’s body. The participant sat on a chair of standard height for working at a desk and facing a digital tablet that was located approximately 40 cm in front of them. The participants were instructed to match the designed criterion time as accurately as possible and also be as accurate as possible to hit the center of the target. The trajectory of the stylus was not shown on the board when performing the task except the cursor that was always visible during the whole trial. The algebraic temporal and algebraic spatial errors from the respective task criterion were each presented numerically on the computer screen immediately (< 2 s) as information feedback after the completion of each trial. Participants were instructed that their performance should be as close as possible to the dual space-time task criteria.

This was not a reaction time experiment and the participants were instructed not to respond to the beep sound as fast as possible. A beep sound was given when participants held the stylus on the home position for 600 ms. He/she was to begin each trial when they were comfortable and ready after the beep sound. The initiation of movement was defined by the stylus crossing the low velocity threshold of 3 mm/s and stayed above that threshold for 30 ms. The stylus was to remain in contact with the tablet during the movement until the trial was completed. The trial was finished when the stylus came to a stop on the target. The movement stop was defined by the velocity of the stylus being below 3 mm/s for greater than 40 ms. The next trial started as soon as the participant returned the stylus back to the home position. A 3–5 min break was provided after each 100 trials.

### Submovement analysis

The movement trajectory was assessed based on the horizontal (X) and vertical (Y) coordinates of the stylus tip on the tablet. If participants failed to complete a trial, those data were excluded from the analysis. The rate of outliers was low (less than 1% overall) and inclusion of missed target trials did not affect the main results. The raw displacement data were low-pass filtered (second-order Butterworth filter, cutoff frequency 5 Hz). Movement velocity, acceleration and jerk were calculated as the first, second and third derivatives of movement displacement, respectively. The movements of target aiming were performed mainly along the X coordinate and only the displacement data recorded on this coordinate were analyzed.

To examine the properties of the submovements, we measured the kinematic structure of the trajectory by implementing the algorithms described by Chua and Elliott [6] to detect the different types of submovements within a trial. Four types of submovements were defined (see Fig 1). The first two types of submovements were related to a significant deviation from the acceleration profile in the period between start of the movement and peak velocity (pre-peak), and also the period between the peak velocity and the end of the movement (post-peak). A search for a reversal point to identify significant deviations in the acceleration profile other than the first peak acceleration was performed. If a reversal point was found, then the subsequent reversal point was marked. Once these two reversal points were identified, two criteria had to be satisfied. First, the amplitude between these two reversal points had to fulfill at least 10% of the maximum absolute amplitude in acceleration. Second, the duration of these two points should equal or exceed 72 ms [6].

The last two types of submovements were: 1) zero crossing of the acceleration profile following peak velocity (negative to positive for undershooting); and 2) identify the reversal of the movement (overshooting). In searching for a negative to positive transition in the acceleration profile, the amplitude of such transition in acceleration corresponds to the velocity profile had to equal or exceed 50 mm/s and also satisfy the temporal criterion of 72 ms. For the movement reversal, a change in the sign of velocity was identified. In addition, the absolute amplitude of negative velocity had to maintain above 10 mm/s and satisfy the temporal criterion (72ms). This algorithm is only one of several methods that have been used to identify submovements (e.g., [8–9, 29]) and was established by using a similar task (e.g., discrete aiming movement) to that used here. We counted the number of respective types of submovement and total number of submovements within a trial. In addition, the incidence of different types of submovement was computed for each condition as the number of trials with respective type of submovement divided by the total number of trials performed in this condition. We contrasted Chua and Elliott [6]’s algorithm with Shmuelof et al. [29]’s algorithm to detect submovements. These approaches yielded similar results for submovements and are reported elsewhere [32].

### Data analysis

Statistical analysis was performed in SPSS Window Version 8.2. Repeated measures ANOVAs were used to examine the effect of the space-time task movement conditions on each dependent variable for the quantity and incidence of each submovement type, respectively. The Greenhouse-Geisser method was used to correct for violations of sphericity and the Bonferoni correction was applied for the post hoc comparisons, with partial *eta* square (η_p_^2^) [33] revealing the effect size. MATLAB Version 8.2 (Mathworks, R2013b) and SigmaPlot Version 10.0 were used for data preparation, analysis, and plotting figures of the dependent variables.

## Results

### Number of submovements

**Fig 2:**
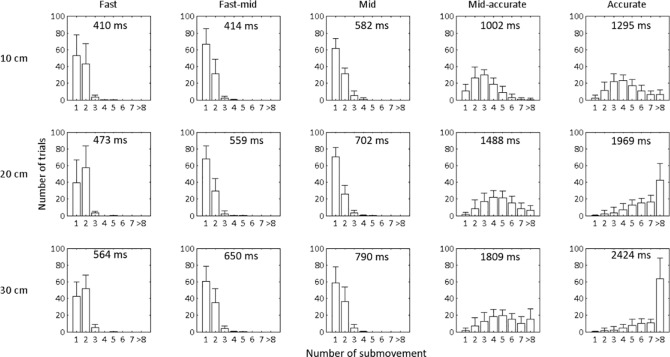
The average number of trials as a function of number of submovements (1 = only primary movement, 2 = primary movement with secondary submovement etc.) in a trial for different movement space-time conditions. Top row indicates 10 cm, middle row shows 20 cm, and bottom row is 30 cm movement amplitude. The error bars represent the between-participant standard deviation. The upper middle of each graph shows the mean movement time for each space-time condition.

**Fig 3:**
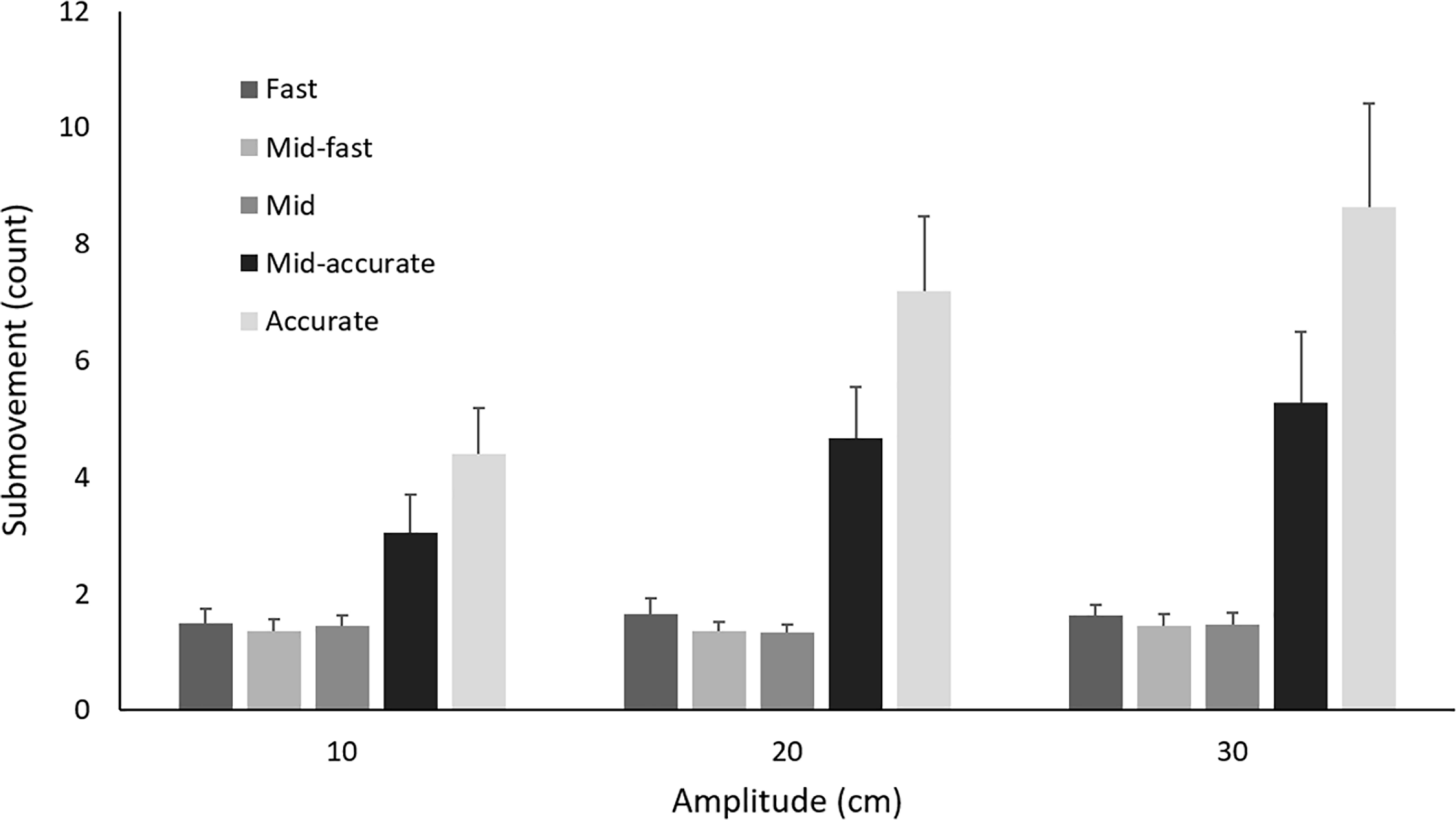
The average number of submovements for 3 different amplitudes as a function of space-time conditions. The error bars represent the between-participant standard deviation.

Fig 2 depicts the total number of trials (average of participants) that showed for a given number of submovements in a trial as a function of the movement space-time condition. The accurate and to a lesser extent the mid-accurate movement conditions had the largest range of the number of submovements occurring within a trial. The distribution of the number of submovements shifted from positive skewness at fast, fast-mid and middle conditions to negative skewness at mid-accurate and accurate conditions. Fig 3 shows the average number of submovements of the discrete aiming task as a function of the space-time constraints at the three movement amplitudes.

The 2 way (3 amplitudes x 5 conditions) repeated measures ANOVA for number of submovements showed that the main effect of amplitude was significant, *F*(2, 22) = 58.20, *p* < .001, η _p_^2^ = .84, where the number of submovements was significantly different from each other at all amplitudes. The main effect of space-time conditions was also significant, *F*(1.18, 13.05) = 224, *p* < .001, η _p_^2^ = .95. The post hoc paired comparisons showed that all space-time conditions were significantly different from each other (*ps* < .05) except the fast from the fast-mid conditions.

The amplitude by space-time conditions interaction was also significant, *F*(2.20, 24.16) = 41.23, *p* < .0001, η _p_^2^ = .78. The post hoc simple main effect analyses showed that the number of submovements at 30 cm was significantly greater than that at 10 cm in the fast and fast-mid conditions, *ps* < .05. Moreover, all amplitudes were significantly different from each other (30 cm > 20 cm > 10 cm) in mid-accurate and accurate conditions, *ps* < .05. In addition, there were significant differences between all the space-time conditions in all of the amplitudes (accurate > mid-accurate > middle > fast-mid = fast), *ps* < .05, except fast from fast-mid and middle conditions in the 10 cm, fast from fast-mid in the 20 cm, and fast from fast-mid in the 30 cm.

### Type of submovements

**Fig 4:**
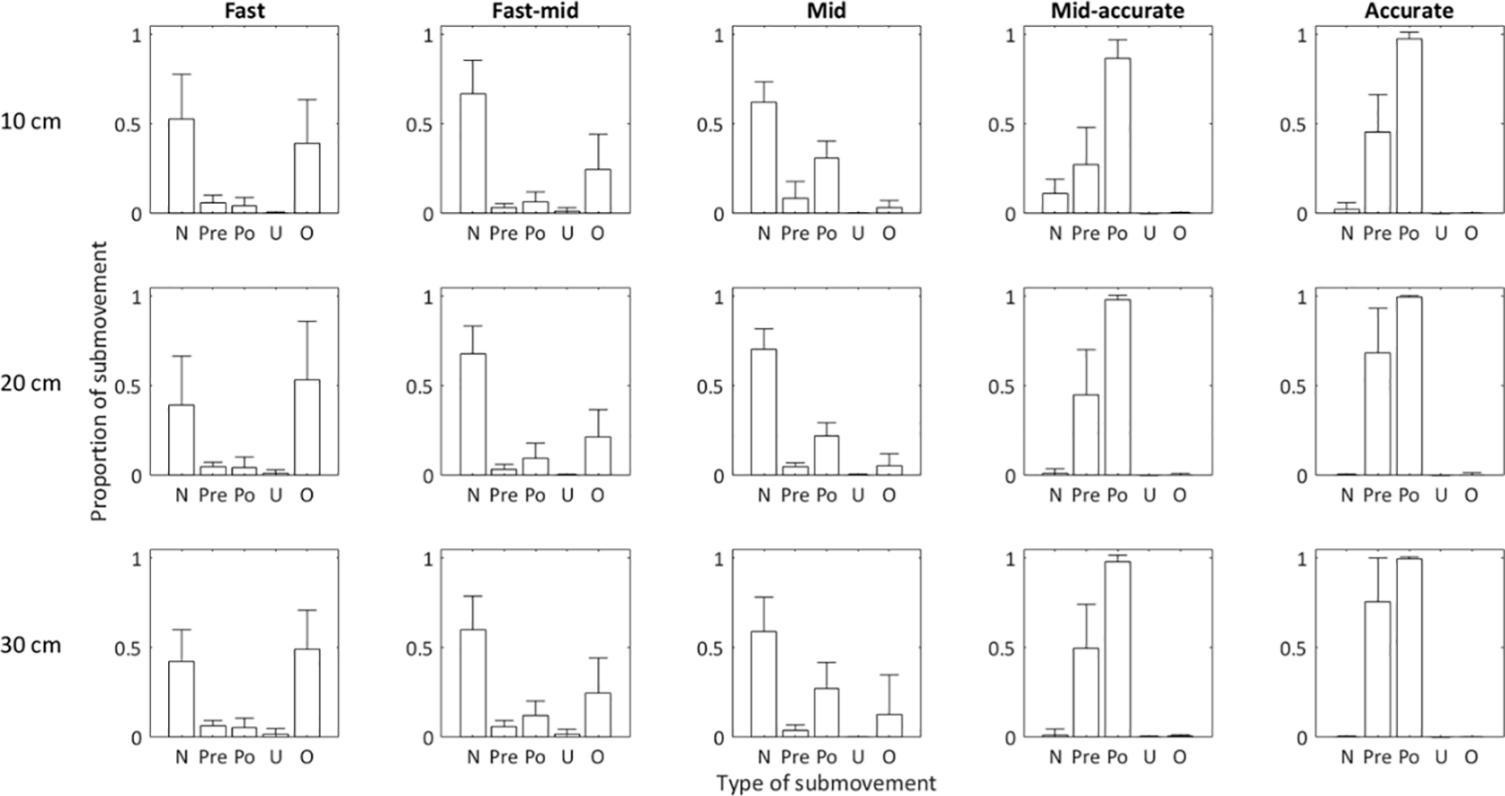
The distributions of different submovement types (N = none, Pr = pre-peak, Po = post-peak, U = undershoot, and O = overshoot) for the 5 space-time conditions (fast, fast-mid, middle, mid-accurate, and accurate). The different rows indicate different movement amplitudes (10, 20 and 30 cm). The error bars are the between-participant standard deviation.

Fig 4 shows the mean proportional incidence of each submovement type across the 15 space-time movement conditions. The figure illustrates that the no submovement (none, only primary movement) and overshooting types were more frequent in the fast and fast-mid conditions. Moreover, the incidence of no submovement and post-peak increased but the incidence of overshooting decreased at middle conditions. Further, the incidence of pre-peak and post-peak increased but the prevalence of no submovement deceased at mid-accurate and accurate conditions. In general, however, undershooting was rare in all space-time movement conditions.

The above differences were all supported by significant type by space-time conditions interactions for 10 cm, *F*(16, 176) = 73.33, *p* < .0001, η _p_^2^ = .87; 20 cm, *F*(16, 176) = 87.45, *p* < .0001, η _p_^2^ = .88; and 30 cm, *F*(16, 176) = 84.19, *p* < .0001, η _p_^2^ = .88, respectively. All three amplitudes showed similar trends of submovement types across the space-time conditions. The post hoc simple main effect analyses (Table 1 for details) showed that the most of the submovement types were significantly different from each other at all space-time conditions and that most of the space-time conditions were significantly different from each other at all submovement types.

**Table 1:** Statistical results for incidence of submovement types (post hoc simple main effect analyses).

Amplitudes	Non / fast	Pre-peak / fast-mid	Post-peak / mid	Undershoot / mid-accurate	Overshoot / accurate
10cm	N: F > MA, AC; FM > MA, AC; M> MA, AC; MA > AC F: N > Pr, Po, U; Pr > U & Pr < O; Po > U & Po < O; U < O	Pr: F < AC; FM < MA, AC; M < MA, AC; MA < AC FM: N > Pr, Po, U, O; Pr < O; Po > U; U < O	Po: F < M, MA, AC; FM < M, MA, AC; M < MA, AC; MA < AC M: N > Pr, Po, U, O; Pr > U, O & Pr < Po; Po > U, O	MA: N > U, O & N< Po; Pr > U, O & Pr < Po; Po > U, O	O: F > M, MA, AC; FM > M, MA, AC AC: N < Pr, Po; Pr > U, O & Pr < Po; Po > U, O
20cm	N: F > MA, AC & F < M; FM > MA, AC; M> MA, AC F: N> Pr, Po, U; Pr < O; Po < O; U < O	Pr: F < MA, AC; FM < MA, AC; M < MA, AC; MA < AC FM: N > Pr, Po, U, O; Pr > U & Pr < O; Po > U; U < O	Po: F < M, MA, AC; FM < M, MA, AC; M < MA, AC M: N > Pr, Po, U, O; Pr > U & Pr < Po; Po > U, O	MA: N < Pr, Po; Pr > U, O & Pr < Po; Po > U, O	O: F > FM, M, MA, AC; FM > M, MA, AC AC: N < Pr, Po; Pr > U, O & Pr < Po; Po > U, O
30cm	N: F > MA, AC; FM > MA, AC; M> MA, AC F: N > Pr, Po, U; Pr < O; Po < O; U < O	Pr: F < MA, AC; FM < MA, AC; M < MA, AC; MA < AC FM: N > Pr, Po, U, O; Po > U; U < O	Po: F < FM, M, MA, AC; FM < M, MA, AC; M < MA, AC M: N > Pr, Po, U, O; Pr > U & Pr < Po; Po > U	MA: N < Pr, Po; Pr > U, O & Pr < Po; Po > U, O	O: F > FM, M, MA, AC; FM > MA, AC C: N < Pr, Po; Pr > U, O& Pr < Po; Po > U, O

N = None, Pr = pre-peak, Po = post-peak, U = undershoot, O = overshoot F = fast, FM = fast-mid, M = mid, MA = mid-accurate, AC = accurate ps < .05

## Discussion

In the present study, we systematically manipulated the space-time constraints of a discrete aiming task to investigate their effect on the probability of particular characteristic types of submovements in trajectory kinematics. The findings showed that performing aiming movements under fast average velocity conditions resulted in a smaller overall number of submovements but a relatively large number of overshooting submovements. On the other hand, the number of submovements in pre-peak and post-peak velocity categories increased when the temporal criteria of the task became longer in duration (e.g., mid-accurate and accurate conditions). The incidence of undershooting submovements, however, was low in all space-time conditions. Overall, the distributional patterns of the prevalence of submovement types were determined by an interaction of the amplitude and time of the movement task conditions, and the constraining influence of average movement velocity.

Most of the participants used predominantly two patterns of submovements in the fast and fast-mid conditions. The reduced movement time was related to a participant’s ability to increase the initial force impulse, but also increased the probability of time-consuming corrections at 10, 20 and 30 cm [3, 9]. Indeed, the relative prevalence of the submovement characteristics observed during the discrete aiming movements is consistent with the original findings of Woodworth [4]. The submovement types can be re-categorized into the two successive general phases, an impulse initiation phase and a current control phase [9, 31].

Given the inherent stochastic properties of neural-motor noise in the human system [3], it has been proposed that corrective submovements are needed to achieve the target goal [10–11]. Consistent with this interpretation, the relatively high movement variability in the spatial dimension under the fast condition may be the result of a control strategy with few submovements to successfully match the time criterion (e.g., [4, 22]). In this view, the participants increased movement speed by trading movement accuracy that reflects the speed-accuracy trade-off phenomenon (see Results in [24]).

Carlton [22] indicated that the participant adopted the single acceleration-deceleration control strategy to match the goal movement time was a result of temporal accuracy constraints. Nevertheless, in the case of considering both spatial and temporal constraints together, the spatial dimension seems to have a greater impact than the temporal dimension in the fast and fast-mid conditions across the three different amplitudes. In this case, participants predominantly produced overshooting submovements (e.g., time- consuming correction) to correct the movement error in the spatial dimension but, at the same time, decreasing the movement accuracy in the temporal dimension (e.g., longer movement time). It is also possible that the dual task requirements may distract performers’ attention to either time monitoring or space monitoring and result in a specific combination of submovement types. Moreover, the best solution for achieving a stringent temporal constraint (e.g., fast condition) would be to find the optimal compromise between more forceful primary submovement with fewer time-consuming submovement [1, 9, 11, 31, 34].

The visually guided submovement of overshooting has been hypothesized to occur following an initial primary impulse or submovement so as to contact the target accurately (e.g., [4, 10, 15, 35–36]). This interpretation is based on the assumption that the movement time is sufficient to allow visual information to be processed (e.g., MT less than 100 ms, [37]). However, overshooting submovements have also been characterized as motion termination based on active and passive origins, such as the third phase of tri-phasic muscle activation and the viscoelastic properties of muscle–tendon complex [18]. Given that we did not manipulate either the availability of visual information or the viscoelastic properties of the limb, our findings do not allow us to distinguish visually and biomechanically based overshooting submovements in discrete aiming.

Elliott and colleagues [6, 31, 34] postulated that the first phase of aimed movement (initial impulse) might not be completely ballistic as Woodworth [4] originally thought. They proposed a multiple-process model of limb control that holds that the initial impulse reflects a more continuous form of online control rather than exclusively a ballistic form [23, 38]. Our results on pre- and post-peak velocity trajectory changes provide support for this interpretation at all of the 3 movement amplitudes. In addition, the incidence of post-peak increased from fast, fast-mid to middle conditions, even though the percentage of incidence was relatively small (e.g., lower than 40%). Indeed, this type of submovement showed significant deviations in the velocity profile (after peak velocity) of the primary aimed submovement. Elliott et al. [39] suggested that any discontinuity in the movement trajectory that occurred short of the target is an undershoot. For them, the acceleration profile did not need to have a zero crossing to consider as a corrective submovement to amend a primary movement when it fall short of the target if uncorrected. Our results in the middle conditions at the all of the 3 amplitudes supported their operational definition and reflected that participants had more time (time criteria of middle conditions: 10 cm, 550 ms; 20 cm, 650 ms; 30 cm, 750 ms) to use visual feedback to adjust movement trajectories, thus contributing to better accuracy and consistency (e.g., [6]).

The movement kinematic profiles showed an asymmetric velocity profile in which participants achieved higher peak velocities earlier to get the limb closer to the target point and spend more time after peak velocity to use visual feedback for limb control [6, 40]. However, the small percentage of target undershoots and post-peak submovements at the three different amplitudes are inconsistent with some previous findings (e.g., [30, 38, 41]). It is possible that the target width we used as essentially a dot (1 mm in diameter as cursor) was too small to induce a similar effect. Nevertheless, it has been shown that target overshoots occur when the target size is small (e.g., [22, 42]).

Fradet et al. [18] proposed that the type of submovements observed during the task are not as homogeneous as traditional interpretations have held [i.e. visually guided corrections– 10], in that different types of submovement emerge under different task constraints (e.g., task mode and target size). Our results are also consistent with this proposal and show that even in the single task of a discrete aiming movement, different distributions of the types of submovements were observed under different spatial-temporal task constraints. And, while average velocity has a strong role in determining the probability of submovement types, velocity interacts with movement time and amplitude in determining the nature and number of submovements.

It was expected that the number of submovements would increase when the task temporal constraint (movement time) increased in duration, especially at 20 and 30 cm. In contrast to the short duration condition, the spatial dimension seems to decrease the impact of submovements and allowed participants to produce numerous time-consuming submovement. In this case, both spatial and temporal constraints can be satisfied by increasing the number of submovement (e.g., achieving the goal movement time) and by reducing the spatial error through visual feedback (e.g., hitting the target).

Indeed, the results showed that the pre-peak and post-peak types were the main sources of submovement for mid-accurate and accurate conditions. These two types of submovements reflect participants using visually and proprioceptive information guided adjustments to produce movement trajectories in a continuous process (e.g., [6]). It is also possible that the high incidence of the pre- and post-peak submovements in the low average velocity movement conditions are due to the inability of muscle contraction to generate a low force steadily and smoothly as Fradet et al., [18] suggested (with motion fluctuations of a stiffening limb system). Milner [8] made a similar observation and concluded that participants used this strategy to better estimate the movement time when they were required to match the time goals. Indeed, these types of submovement have been hypothesized as the elementary unit of motor execution [29, 43]. Therefore, the characteristics of submovements may be related to the given set of space-time constraints reflecting the emergent property of the task-individual interaction (e.g., [18, 44]).

The different spatial/temporal constraints are related to the trade-off in movement variability when space and time dimensions are considered together [24, 45]. Thus, the prevalence of the types of submovements can be considered a by-product of the movement properties of amplitude and time in the context of the speed-accuracy trade-off (e.g., [2–3]). Indeed, Wright and Meyer [21] pointed out that precisely timed movements are composed of a single pair of opposing force impulses that minimize temporal but not spatial movement variability. Whereas, spatially precise movements are composed of a pre-programmed series of overlapping force impulses that increase the temporal movement variability.

The force-time characteristics of force output have been proposed as the main factor that determines movement variability [3, 46–47]. The mechanisms of motor unit recruitment, such as number of units and discharge rate, have been suggested as contributors to influence variability of the motor output [18, 48–50]. It is possible that moving fast by using only one or two submovements would recruit a larger number of motor units being activated simultaneously in a given time. In addition, there might be a limited time for an impulse to be generated (e.g., 70 ms) [51–52]. In this situation, the force-time summation of the units (e.g., impulse) might be greater than required to meet the task criteria thus causing a high spatial movement error but low temporal movement error (e.g., fast and fast-mid conditions). On the other hand, moving slowly by using many submovements would recruit a small number of force units being activated that overlap in a long time duration causing a low spatial movement error but high temporal movement error (e.g., mid-accurate and accurate conditions). In addition, the sequential activation and deactivation of motor units during slow movement may cause small discrete steps in force level, and probably induce noisy error to movement amplitude.

In summary, the number of submovements within a trial increased from fast (1 or 2 submovements) to slow (7 or more submovements) movement conditions. In addition, the characteristic types of submovement changed from no movement correction and overshooting at fast and mid-fast conditions to pre-peak and post-peak overlapped with increasing and decreasing movement velocity at the mid-accurate and accurate conditions. Changing the spatial-temporal constraints with the same task mode resulted in the presence of different distributional properties of submovements and hence the use of a qualitatively different integration of multiple control processes in movement execution [16]. We have shown that there are qualitatively different distributional patterns of submovement types in discrete aiming tasks that are organized by the quantitative scaling of the average movement velocity arising from multiple control processes to meet the specific space-time task constraints.

## Supporting information

S1 Dataset Spreadsheet presenting number of submovements for each individual in five space-time conditions. (XLSX)

S2 Dataset Spreadsheet presenting type of submovements for each individual in five space-time conditions. (XLSX)
